# Vacuum-Compatible Electrode-Free Poling of PVDF Films Using Glow-Discharge Plasma

**DOI:** 10.3390/polym18151926

**Published:** 2026-08-05

**Authors:** Bogdan A. Basov, Evgeniya L. Buryanskaya, Kamila T. Makarova, Artur R. Zinnatullin, Konstantin M. Moiseev, Alexey S. Osipkov, Alexander A. Maltsev, Bogdan A. Parshin, Dmitriy S. Ryzhenko, Mstislav O. Makeev

**Affiliations:** 1 Laboratory of Ferroelectric Polymers, Bauman Moscow State Technical University, 105005 Moscow, Russia; basovba@bmstu.ru (B.A.B.); buryanskayael@bmstu.ru (E.L.B.); mkt@bmstu.ru (K.T.M.); zinnatullin@bmstu.ru (A.R.Z.); k.moiseev@bmstu.ru (K.M.M.); aam.0205@yandex.ru (A.A.M.); parshbgal@bmstu.ru (B.A.P.); dsr@bmstu.ru (D.S.R.); m.makeev@bmstu.ru (M.O.M.); 2 Department of Materials Science of Semiconductors and Dielectrics, National University of Science and Technology MISIS, 119049 Moscow, Russia; 3 Department of Electronics of Organic Materials and Nanostructures, N.M. Emanuel Institute of Biochemical Physics (IBCP), Russian Academy of Sciences (RAS), 119334 Moscow, Russia

**Keywords:** ferroelectric polymers, PVDF, piezoelectricity, phase transition, glow-discharge plasma

## Abstract

Glow-discharge plasma (GDP) poling is revisited as an electrode-free method for activating piezoelectricity in poly(vinylidene fluoride) (PVDF) films. Although this method was proposed several decades ago, its effect on the properties of PVDF films has remained poorly understood. In this work, we demonstrate that GDP enables efficient poling of oriented PVDF films without pre-deposited electrodes and investigate the relationship between plasma treatment time, structural evolution, and piezoelectric response. Commercially available 25 μm-thick oriented PVDF films (PolyK) were treated in a DC glow discharge for 15 s to 15 min and characterized using FTIR, DSC, piezoresponse force microscopy, UV–Vis–NIR spectrophotometry, quasi-static *d*_33_ measurements and water contact-angle measurements. GDP poling produced a side-averaged piezoelectric coefficient *d*_33_ of up to ~25 pC/N within 1–5 min, with local maxima at approximately 1, 2.5, and 5 min. This behavior was accompanied by pronounced changes in the domain structure, including an increase in the ferroelectric domain size from 86 to 552 nm, while the crystallinity and electroactive phase fraction changed only moderately. Plasma treatment also increased the wettability of the plasma-facing surface, reducing the water contact angle from about 85° to 42° within 3 min. At longer treatment times (>5 min), however, the piezoelectric response decreased and the optical transparency deteriorated because of increased haze and turbidity, most likely associated with plasma-induced chemical modification of the surface layers. These results indicate that GDP poling has an effective processing window of 1–5 min. The proposed approach provides a vacuum-compatible and electrode-free route for preparing PVDF films with increased surface wettability for flexible piezoelectric sensors, wearable electronics, and integrated polymer-based devices, because it is compatible with electrode deposition on an already activated polymer surface within a single vacuum cycle.

## 1. Introduction

Flexible polymer films based on polyvinylidene fluoride (PVDF) and its copolymers have been the subject of intensive research since the discovery of their ferroelectric properties in 1969 [1,2]. Today, these materials represent the most technologically mature class of ferroelectric polymers and are widely employed in sensor applications owing to their unique combination of transparency, flexibility, mechanical strength [3], biocompatibility [4], and high piezoelectric and flexoelectric coefficients relative to other polymer ferroelectrics [5]. A key advantage is their broad operational frequency range and the close acoustic impedance match of PVDF copolymers with liquids and biological tissues, making them particularly well suited for vibration sensors and hydrophones [6,7]. Among the most promising applications of PVDF-based ferroelectrics are medical and biomedical devices, including ECG [8] and oxygenation sensors [9], photoacoustic transducers for microscopy [10] and angiography [11], and ultrasound imaging systems [12,13,14], as well as emitters and sensors for transcranial ultrasound therapy [15]. The continuing expansion of flexible electronics, wearable healthcare devices, and high-frequency ultrasonic systems has further increased the demand for scalable and reproducible manufacturing routes for PVDF-based sensor elements.

The technological process of manufacturing sensitive elements of such sensors, which are ferroelectric polymer films with electrodes applied to their surface, includes formation of the film itself (most often by casting [16,17,18] or extrusion [19,20,21]), its subsequent mechanical stretching [22,23,24], and annealing [25]. These stages collectively determine the dielectric strength of the PVDF film [26], which is a critical parameter for the final poling step. In contrast to piezoceramics, whose properties are predominantly determined by chemical composition, mechanical stretching and annealing have been shown to significantly influence the structure, phase composition, and, consequently, the electroactive properties of PVDF and its copolymer materials [22].

The poling of ferroelectric materials involves applying a strong electric field to align the randomly oriented dipoles within the film’s domain structure, thereby enhancing its piezoelectric properties [16]. The two conventional techniques for this purpose are contact poling [27] and corona discharge poling [28]. Contact poling is well suited to laboratory studies because it is well established and can be implemented using standard equipment. Its primary drawbacks, however, are the necessity for preliminary electrode deposition—a limitation for roll-to-roll manufacturing—and a significant risk of dielectric breakdown, which can result in complete sample failure. Similarly, corona discharge poling, which usually operates at fields near the breakdown threshold, suffers from high field inhomogeneity. This limitation not only increases the risk of film breakdown [29] but can also lead to structural degradation and premature termination of the poling process.

This study investigates a relatively understudied poling method that utilizes glow-discharge plasma (GDP). This approach was first proposed by McKinney et al. in 1980 [30]. However, GDP poling attracted little subsequent research attention, presumably due to the considerable technological challenges it presented. While the poling mechanism of GDP is expected to be similar to that of corona discharge, as both rely on the accumulation of surface charge to orient dipoles in the ferroelectric film, GDP offers several important advantages. The use of flat electrodes enables the generation of an electric field with higher spatial uniformity across the film area compared with conventional corona discharge systems [31]. In addition, the vacuum environment required to sustain glow discharge provides a clean processing atmosphere, improves process reproducibility, and enables subsequent electrode deposition on the activated polymer surface within a single vacuum cycle without exposure to ambient contaminants.

For flexible piezoelectric sensors and wearable electronic devices, the poling stage is not only a means of activating the piezoelectric response but also a critical technological step that affects process scalability, electrode configuration, film integrity, and compatibility with subsequent device fabrication. In addition to poling itself, the quality of the polymer–electrode interface is often a limiting factor for PVDF-based devices because fluoropolymers possess low surface energy and exhibit poor adhesion to metallic coatings. Plasma treatment has been shown to increase the surface energy of PVDF and improve the adhesion and quality of subsequently deposited metallic layers [32,33,34,35].

Another important aspect is the preservation of the activated polymer surface. Exposure of plasma-treated polymers to ambient atmosphere may result in the adsorption of moisture, airborne contaminants, and organic species, which may change the surface chemistry and affect the quality of the polymer–electrode interface [36]. In conventional corona poling, the treated film is exposed to air prior to subsequent processing steps, whereas GDP processing allows poling and subsequent electrode deposition to be performed within the same vacuum cycle.

Development of electrode-free and vacuum-compatible poling routes is important not only for activating piezoelectricity but also for improving process integration, interface quality, and compatibility with subsequent device fabrication. Such an integrated approach may be particularly attractive for flexible electronics, ultrasonic sensors, and hydrophones operating under demanding conditions. In these applications, the durability and reliability of electrodes can strongly affect device performance, especially under cyclic mechanical loading or high acoustic pressures.

To further advance GDP poling as a practical processing technique, it is necessary to understand how plasma treatment affects the structure of PVDF and, consequently, its functional properties. The efficacy of the poling process, and thus the resulting macroscopic polarization and piezoelectric response, is governed by the material’s structural features and the nature of molecular mobility in both its crystalline and amorphous regions [37]. Importantly, poling itself induces structural changes in the polymer. For instance, the application of a strong electric field can lead to the propagation of kinks along PVDF molecular chains [38]. Such structural and property modifications in PVDF-based materials have been well documented for traditional poling techniques, such as corona and contact poling, which have been studied for over five decades [39,40,41,42]. In contrast, the specific structural transformations that occur in polymer ferroelectrics during poling via glow-discharge plasma remain largely unexplored.

Unlike previous studies that mainly reported the feasibility of plasma poling [43,44], this study focuses on the time-dependent relationship between plasma treatment, structural evolution, optical degradation, piezoelectric response, and film surface wettability. By combining *d*_33_ measurements with FTIR, DSC, PFM, UV–Vis–NIR spectrophotometry, and water contact-angle measurements, we identify an optimal processing window of 1–5 min in which a high piezoelectric response of approximately 25 pC/N is achieved without pronounced optical degradation. The results establish GDP poling as a controllable and scalable processing route for PVDF films intended for flexible piezoelectric sensors and polymer-based electronics.

## 2. Materials and Methods

### 2.1. Samples

Samples of the oriented (stretched) polyvinylidene fluoride (PVDF) film, 25 μm thick, PVDF-B0040 (PolyK, State College, PA, USA) were used in the poling experiments. According to the manufacturer data, the side-averaged piezoelectric coefficient *d*_33_ after poling should be 22–24 pC/N. Those values were taken as reference values.

### 2.2. Glow-Discharge Plasma Poling Methodology

**Figure 1:**
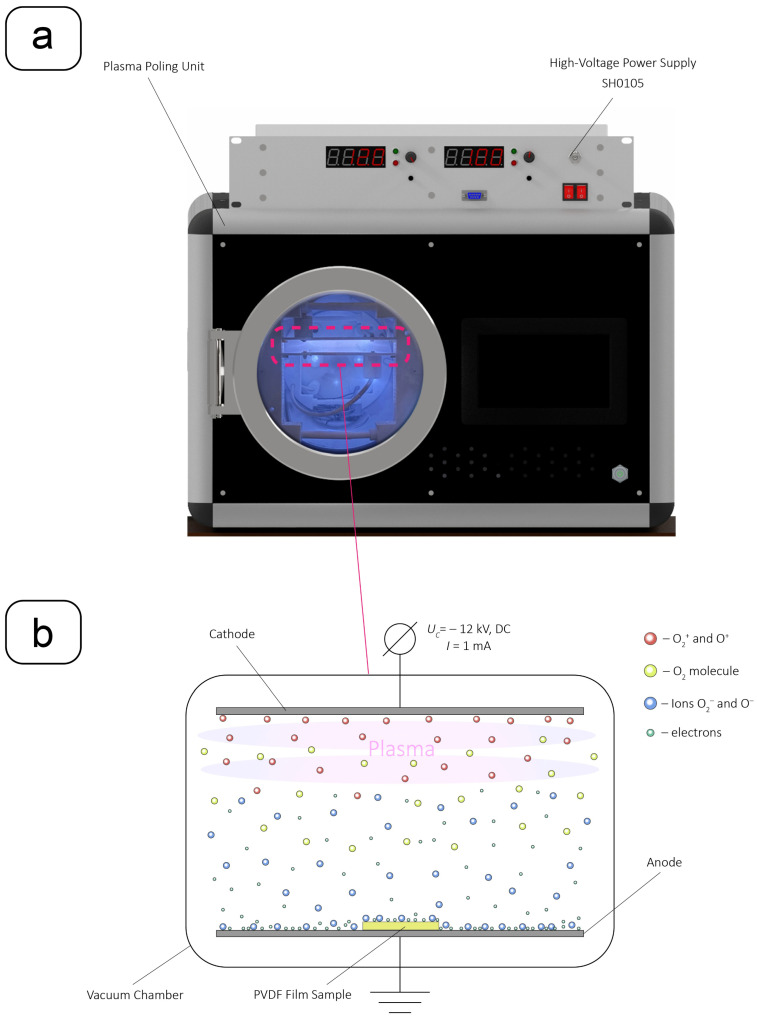
Plasma poling in a DC glow discharge. (**a**) Photograph of the vacuum plasma poling setup. (**b**) Schematic of the discharge and charge deposition on a PVDF film placed on a grounded anode, illustrating the formation of surface charge by electrons and negative ions (predominantly O_2_^−^), which generates a high internal electric field and orients dipoles in the film. Typical conditions: *p* = 2 Pa, *d* = 40 mm, *U* = 12 kV (DC), and *j* = 0.1 A/m^2^.

Plasma poling of the PVDF film samples without the pre-applied electrodes was performed in a vacuum plasma setup modified for the plasma poling tasks (Figure 1a). In each poling process, three PVDF film samples measuring 20 × 20 mm were positioned on the anode (Figure 1b).

GDP poling was carried out at a residual pressure of 2 Pa, an interelectrode gap of 40 mm, a discharge voltage of 12 kV, and a current density of 0.1 A/m^2^. The treatment time ranged from 15 s to 15 min.

The mechanism of the PVDF poling process in the glow-discharge plasma lies in the generation of the negative O_2_^−^ ions and electrons in the high-voltage direct current (DC) glow discharge. Such generated species interact with the surface of the PVDF film positioned on a grounded anode (see Figure 1b) [45]. By analogy with corona poling of PVDF films and plasma-induced charging of dielectric surfaces, these species can create a virtual electrode, resulting in the formation of a potential difference across the film thickness during the poling process [46]. The possibility of surface charge accumulation during treatment in DC glow-discharge plasma has also been experimentally demonstrated in [47].

In the present work, the potential to which the PVDF film surface is charged during plasma poling was not measured experimentally. However, based on the literature data, we assume that, as in the case of corona poling, this potential tends toward a value close to the discharge potential and the electric field across the film thickness should be considered an estimate based on the proposed surface-charging mechanism rather than a directly measured value.

### 2.3. Characterization Methods

#### 2.3.1. Piezoelectric Measurements

Piezoelectric response of the GDP-poled samples was measured using the quasi-static Berlincourt method with the *d*_33_-meter YE2730A (Sinocera Piezotronics, Yangzhou, China) at the calibrated load of 0.25 N and frequency of 110 Hz at 12 points on each side of the film.

#### 2.3.2. Water Contact-Angle Measurements

To evaluate the effect of treatment in a high-voltage glow-discharge plasma in a residual gas atmosphere on the wettability of the PVDF film surface facing the plasma during poling, water contact-angle measurements were performed. Deionized water droplets with a volume of 3 μL were deposited onto the surface of PVDF film samples using a manual microsyringe 5 min after plasma poling. Droplet profiles were recorded in side-view geometry using a laboratory optical microscope equipped with a ToupTek UCMOS10000KPA digital camera (Hangzhou, China). The obtained images were analyzed using ImageJ software (version 1.54t, National Institutes of Health, Bethesda, MD, USA) and the DropSnake plugin (accessed on 7 July 2026) according to the procedure proposed by Stalder et al. [48]. For each sample, four contact-angle measurements were performed at different surface points. For each droplet profile, the left and right contact angles were determined and then averaged. Based on the obtained data, the mean contact angle and the confidence interval at a confidence level of 95% were calculated for each experimental point.

#### 2.3.3. FTIR Spectroscopy

Phase composition of the PVDF film samples was determined using the Fourier transform infrared spectroscopy in transmission mode. Transmission measurements were performed using the PerkinElmer 1760X FTIR spectrometer (Waltham, MA, USA) in the wavenumber range of 420–4000 cm^−1^. However, the transmission spectra were quantitatively analyzed only in the range of 420–850 cm^−1^, since optical density of the films of the studied thickness exceeded 2.0 in the 860–1400 cm^−1^ range. The entire primary spectral treatment was performed using the Bruker OPUS 7.2 software (Bruker Corporation, Bremen, Germany).

ATR-FTIR spectra of the samples were obtained with FTIR spectrometer FT-803 (Simex, Moscow, Russia) with diamond single-reflection ATR unit. ATR-FTIR spectra were collected in the range of 550–1800 cm^−1^ with a resolution of 2 cm^−1^.

The film phase composition (electroactive phase fraction and α/am-phase relation) was computed using the transmission FTIR spectroscopy based on the literature data [49].

Electroactive phase fraction:

(1) $$F_{ea}=\frac{A_{837}}{1.26\times A_{763}+A_{837}},$$

where *A*_763_ and *A*_837_ are the amplitudes of peaks centered at 763 and 837 cm^−1^ (*α*-phase and electroactive (*β* and *γ*) phase peaks), respectively.

The *α*-phase content in film volume was calculated from transmission mode FTIR spectra (in transmission mode the amorphous phase band 602 cm^−1^ has relatively stable intensity for all investigated samples, whereas *α*-phase band 614 cm^−1^ differs for abovementioned samples; in ATR mode, both bands were insufficiently stable for quantitative calculations). The relation between *α* and *am* phases is calculated according to the formula:

(2) IαIam=A614A602,

where *A*_614_ and *A*_602_ are the amplitudes of peaks centered at 614 and 602 cm^−1^ (*α*-phase and *am* (amorphous) phase peaks), respectively.

Amplitudes of all peaks were computed after deconvolution using the PeakFit 4.12 (Systat) software. The peak shape was assumed to be Gaussian, and the peak center coordinates for curve computation were refined using the literature data.

#### 2.3.4. Piezoresponse Force Microscopy

Ferroelectric properties and morphology alterations were studied by the scanning probe microscopy (SPM) methods using the NTEGRA Prima atomic force microscope (NT-MDT SI, Zelenograd, Russia) with cantilevers coated with the FM*G*01/Pt platinum conductive coating (Tipsnano, Tallinn, Estonia). Experimental data were processed using the Gwyddion 2.67 software (Czech Metrology Institute, Brno, Czech Republic).

SPM was used to obtain film surface topographies and compute the root-mean-square (RMS) roughness values for glow-discharge plasma-poled samples of PVDF film. In the piezoresponse force microscopy mode, vertical and lateral piezoresponse signal distribution maps were obtained, and measurements were carried out at the fixed frequency of 120 kHz and voltage of 10 V. For each sample, at least 3 independent PFM images acquired from different regions were analyzed.

The characteristic domain size *ξ* was found by computing the correlation function of the vertical piezoresponse signal distribution depending on the *r* coordinate:

(3) Cr = A×exp–rξ2h, 

where *A* is the constant; *r* is the distance from the central peak (nm) determined from the autocorrelation function image; *ξ* is the average value of the domain size (nm); and *h* (0 < h < 1) is the parameter [50,51].

#### 2.3.5. Differential Scanning Calorimetry

The samples’ thermal properties and crystallinity degree were determined using the differential scanning calorimetry (DSC) with the NETZSCH DSC 204 F1 Phoenix device (NETZSCH-Gerätebau GmbH, Selb, Germany). For measurements, the samples were positioned in the aluminum crucibles and heated in the temperature range of 25–200 °C at the rate of 10 K/min under argon.

Using the DSC curves made it possible to compute the film crystallinity degree, applying Formula (4):

(4) $$\chi_{c}\;=\;\frac{∆H_{m}}{\left(a\times ∆H_{m}^{\alpha }+b\times ∆H_{m}^{\mathrm{ea}}\right)},$$

where ∆*H_m_* is the film melting enthalpy; ∆*H^α^_m_* is the melting enthalpy of the α-phase, which is 93.07 J/g; and ∆*H^ea^_m_* is the melting enthalpies of the electroactive (equal for β- and γ-phase), respectively), which is equal to 103.4 J/g [52,53].

#### 2.3.6. UV–Vis–NIR Spectrophotometry

Transmittance coefficients (T(λ)) of the film samples were measured in the range from 350 to 1000 nm using the two-beam spectrophotometer UV-3600i Plus (Shimadzu, Kyoto, Japan). To distinguish the film absorption and scattering effect, two optical configurations were used for measuring the transmittance: free space (*T_direct_*) with the slit width of 8 nm and integrating sphere (*T_sphere_*) with the slit width of 32 nm. In all the experiments, the slit spectral resolution was 5 nm. Total luminous transmittance (*τ_t_*) for the standard light source CIE D65 was computed according to the method provided in ISO 13468-2:2021 [54]. Haze, H(λ), was computed using Formula (5) according to the method described in ISO 14782:2021 [55]:

(5) $$H\left(\lambda \right)=\frac{T_{sphere}\left(\lambda \right)-T_{direct}(\lambda )}{T_{sphere}(\lambda )},$$

where *T_sphere_*(*λ*) is the transmittance coefficient measured with the integrating sphere; *T_direct_*(*λ*) is the transmittance coefficient measured in the free-space configuration; and λ is the wavelength (nm).

## 3. Results

### 3.1. Piezoelectric Properties

**Figure 2:**
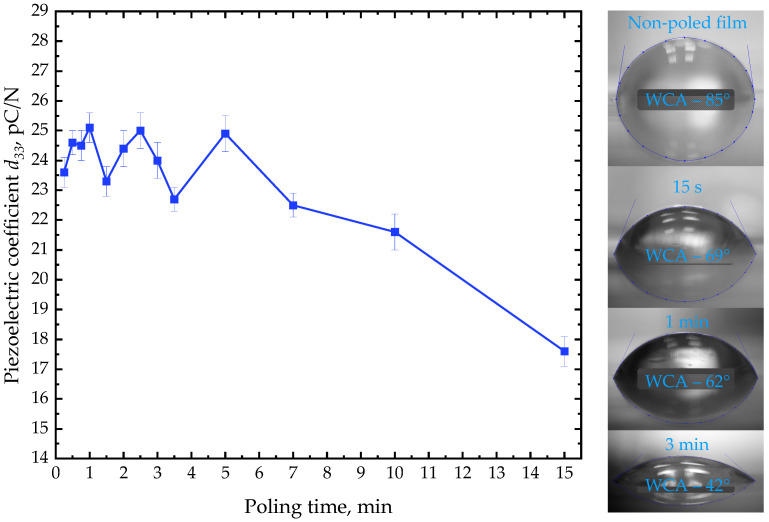
Dependence of the side-averaged piezoelectric coefficient *d*_33_ of PVDF films on the glow-discharge plasma poling time. Representative water contact-angle images are shown for selected treatment times.

Figure 2 shows the dependence of the side-averaged piezoelectric coefficient *d*_33_ on the poling time, together with representative water contact-angle images of the PVDF film surface for the non-poled film and selected samples poled from 15 s to 3 min.

The dependence reveals three maxima in *d*_33_ after GDP poling for 1, 2.5, and 5 min: 25.1, 25.0, and 24.9 pC/N, respectively. This behavior indicates that structural changes occurring during plasma poling affect the piezoelectric response of the PVDF film. After 7 min of treatment, a gradual degradation of the piezoelectric response is observed.

The results of contact-angle measurements on the surfaces of PVDF film samples facing the plasma during poling confirm an accompanying increase in the wettability of the PVDF film surface during plasma poling. Before plasma poling, the water contact angle of the PVDF film was 80–90°. During the first 15 s of poling in residual gases, the contact angle sharply decreased and was in the range of 64–73°. After 3 min of poling, a more pronounced increase in surface wettability was observed: the contact angle was in the range of 39–45°.

### 3.2. Fourier Transform Infrared Spectroscopy

**Figure 3:**
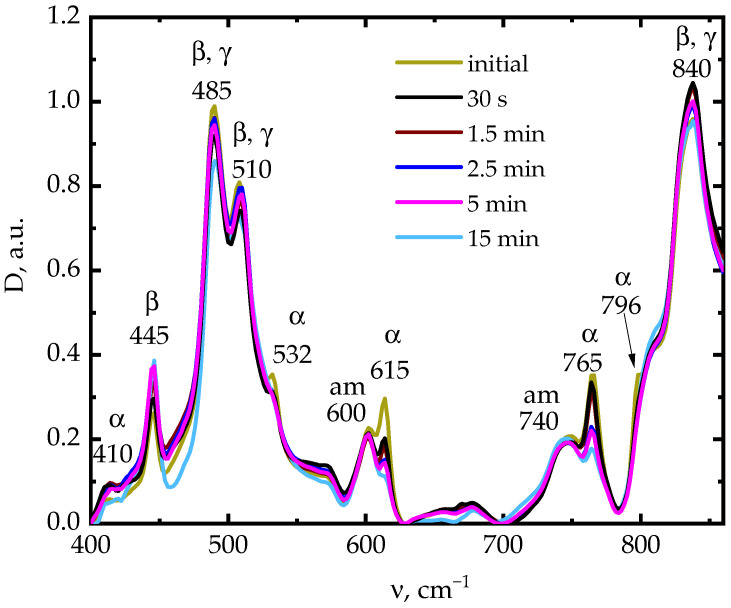
FTIR spectra in the transmission mode for the poled films at different times.

Figure 3 shows the Fourier transform infrared spectroscopy spectra obtained in the transmission mode. For clarity, curves for several poling times are presented.

As can be seen from the data in Figure 3, the *α*-phase proportion decreases during plasma poling. This is evidenced by decreasing peak intensities at 532 cm^−1^, 615 cm^−1^ and 765 cm^−1^. This trend intensifies with an increase in the treatment time, i.e., peaks characteristic of the *α* phase (615 and 765 cm^−1^) for the film poled for 15 min are noticeably lower than the peaks for the film poled for 0.5 min and 1.5 min. Moreover, the intensity of the peak at 445 cm^−1^, which is associated with the β phase, increases as a result of plasma treatment. Other peaks related to the electroactive phase (*β* + *γ*) are changing less noticeably.

The electroactive phase content in the films was computed using the above method (1). The computed values are presented in Table 1.

According to Table 1, the electroactive phase fraction (*β* + *γ*) drops to ~69% within the first minute of poling and then increases to ~79% by 3 min, followed by non-monotonic variations at longer times. The *α*/*am* peaks ratio exhibits the opposite trend. This alteration in the phase composition can be attributed to polymer recrystallization induced by GDP, and incorporation of the amorphous phase chains into the ferroelectric domains formed under the field action.

**Figure 4:**
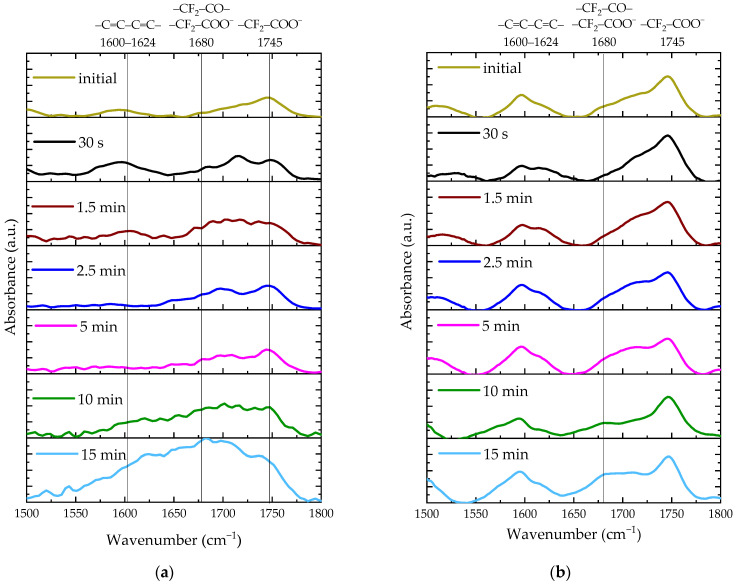
FTIR spectra in the 1550–1800 cm^−1^ region recorded in ATR mode (**a**) and transmission mode (**b**).

It is important to note that treatment of the samples in glow-discharge plasma also changes the IR spectra in the 1550–1800 cm^−1^ range. Figure 4 shows that the intensity of the bands of multiple bonds and carbonyl compounds increases with increasing plasma treatment time.

These changes are primarily observed for bands characteristic of terminal carboxyl groups (1680 cm^−1^) and fluorinated ketones (1720 cm^−1^). This trend is more pronounced in the ATR spectra (Figure 4a), which characterize changes occurring at the surface and in the near-surface layer. Enhancement of the carbonyl group peak (1680 cm^−1^) in the film bulk (Figure 4b) becomes noticeable only at long treatment times.

### 3.3. DSC Results and Crystallinity

**Figure 5:**
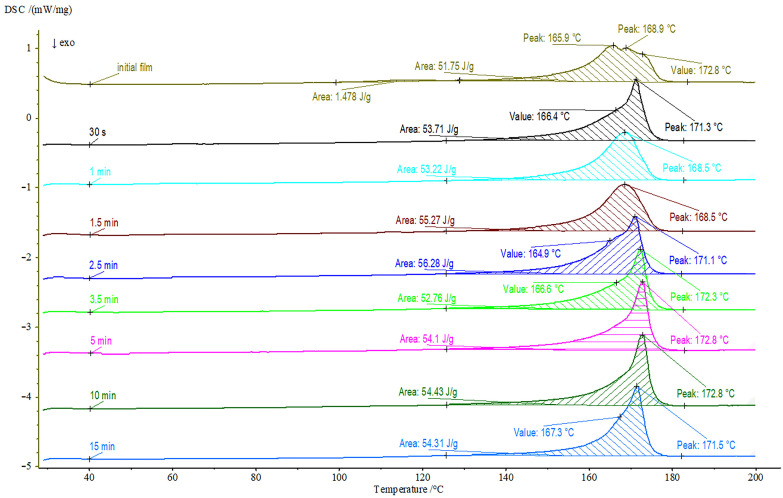
DSC curves for PVDF films: first heating for film with different poling times.

Differential scanning calorimetry made it possible to analyze the samples’ structure. The film’s crystallinity degree was computed based on melting enthalpies of the first heating curves (Figure 5). In addition, phase composition of the films poled using GDP for different times was assessed using the DSC data.

Table 2 presents data for the films’ melting enthalpy and crystallinity degree, calculated with the support of FTIR data.

Plasma treatment significantly affects the shape of the first heating curves. Initially, a sharp peak is observed in a film poled for 30 s, which can be associated with the *α*-phase melting temperature of 171.3 °C. A broad peak at 168 °C is also visible on this curve, which could be attributed to melting of the *β*-phase crystallites. With the increasing poling time (1, 1.5), the peaks merge, indicating possible recrystallization under GDP influence. It is also worth noting that a decrease in the *α*-phase and a nearly constant level in the amorphous phase proportion are observed at these times according to the IR-spectroscopy data (Figure 4). The peak 602 cm^−1^ of amorphous phase appears to be stable because of the very low extinction coefficient in comparison to the extinction coefficient of α-phase. With a further increase in the treatment time, peaks at 168 °C and 171 °C again appear.

The DSC results for the pristine film and the film poled using GDP for 2.5 min reveal a slight increase in the melting enthalpy after glow-discharge plasma poling, from 51.8 to 56.3 J/g, indicating a moderate increase in the overall crystallinity.

The main melting endotherm becomes more pronounced and shifts to higher temperatures after plasma treatment (peak near ~171 °C), suggesting improved crystal perfection and a higher fraction of more stable crystallites [56].

According to Table 2, crystallinity degree depends nonlinearly on the time. The maximum crystallinity degree of 54.9–55.7% is observed for a film poled for 1.5 and 2.5 min. After this, the crystallinity degree decreases and then fluctuates around ~52.3–53.6%. The observed local minima and maxima are also characteristic of the electroactive phase polarization proportion in a sample and the piezoelectric coefficient.

### 3.4. Scanning Probe Microscopy

The glow-discharge plasma influence on the film morphology and domain structure was studied using the piezoresponse force microscopy. Table 3 presents values of the films’ root-mean-square roughness and the ferroelectric domain sizes.

**Figure 6:**
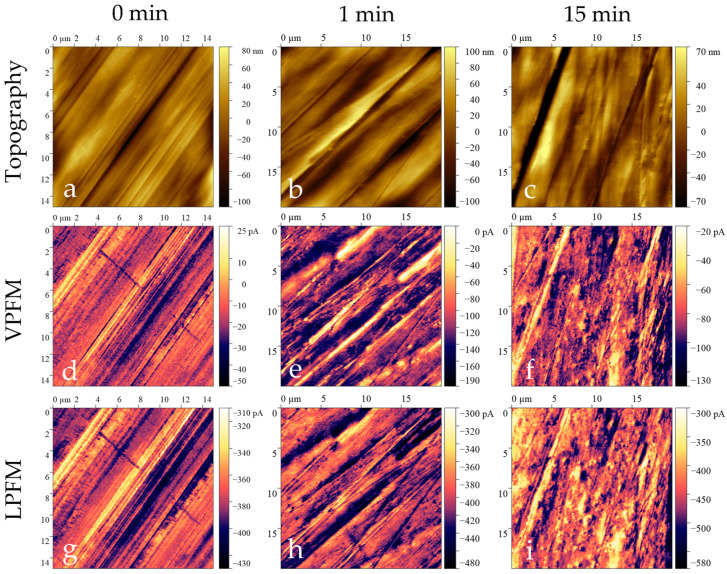
PFM data, where (**a**,**d**,**g**) are the topography, and the vertical and lateral piezoelectric response signals for the original film, respectively; (**b**,**e**,**h**) are those for the film poled for 1 min; (**c**,**f**,**i**) are those for the film poled for 15 min.

Table 3 shows that GDP treatment is insignificantly influencing the film root-mean-square roughness. The RMS values are changing spontaneously, which could be due to the presence of defects and surface inhomogeneity in the original untreated film. The fact that surface roughness according to the AFM data is not changing significantly after plasma treatment could indicate that surface modification is primarily chemical rather than topographic in nature (Figure 6a–c). Uniform plasma treatment could result in homogeneous etching, which creates no new pronounced peaks and valleys, but only slightly smooths the existing irregularities at a level not exceeding the original roughness.

Glow-discharge plasma poling has a significant effect on the polymer domain structure. The PFM data show that the vertical and lateral piezoresponse signal maps change depending on the processing time. For the original film, the piezoresponse signals are partially topographic in nature (Figure 6d,g). Domain size for the original film is minimal. After processing, the domain structure becomes more pronounced, and the piezoresponse intensity increases.

**Figure 7:**
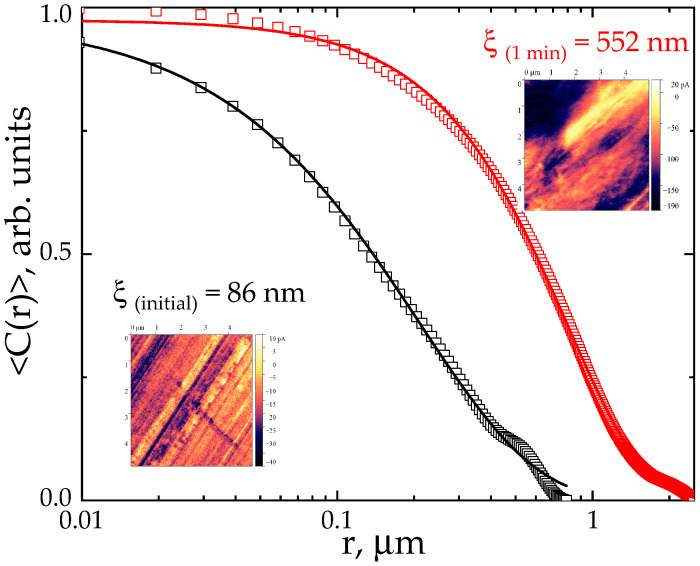
Autocorrelation functions of the ferroelectric domain structures in the initial film (black) and the film poled for 1 min (red).

Figure 7 presents the autocorrelation function for the original and poled films.

GDP poling strongly and non-monotonically affects the ferroelectric domain size. Within the first minute of treatment, the domain size increases from 86 nm to 552 nm; at 1.5 min it drops to 140 nm, followed by a second local maximum of 473 nm at 2.5 min. For processing times ≥3.5 min, the domain size stabilizes in the range of ~100–210 nm. This suggests a complex, staged rearrangement of the domain structure during poling.

### 3.5. Optical Properties

**Figure 8:**
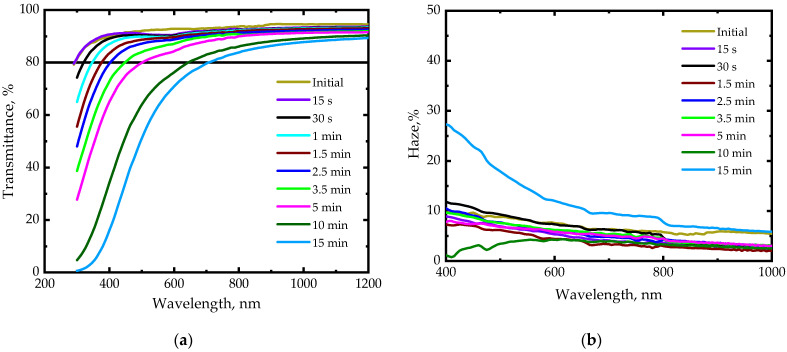
Transmittance with the integrating sphere (**a**) and haze of the initial and plasma-poled films (**b**).

**Figure 9:**
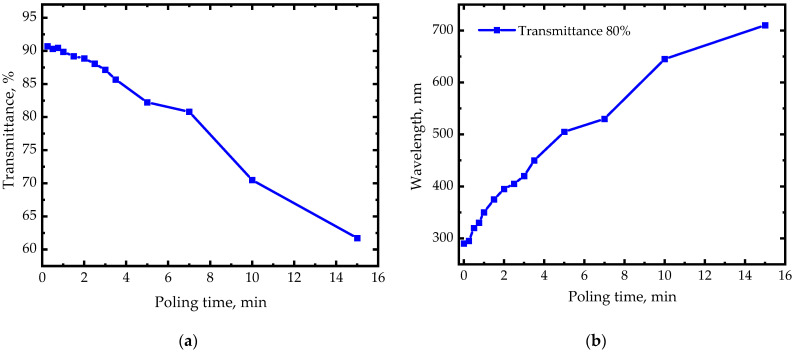
Dependence of the visible-range transmittance calculated using the CIE D65 illuminant (**a**) and the wavelength corresponding to 80% transmittance (**b**) on the poling time.

Poling using the glow-discharge plasma significantly changes the PVDF film optical properties. Film transparency significantly deteriorates with the increasing GDP poling time, and the transmittance coefficient decreases from 90% to 63%. Moreover, films poled in plasma for less than 4 min show minor alterations in their transparency, and the transmittance coefficients decreased by up to ~5 percentage points. Figure 8a,b show transmittance and haze in UV–Vis–NIR (350–1000 nm) wavelength ranges of plasma-poled samples respectively. The dependence of transmittance in the visible range of plasma-poled samples on poling time is shown in Figure 9a. In addition, the spectrophotometric measurements indicate that the initial film has a narrow absorption peak in the near UV region, but it broadens with increasing poling time. Figure 9b shows the dependence of the wavelength corresponding to the 80% transmittance for the PVDF films.

The absorption peak shift towards the long-wave region could be related both to the observed increase in film turbidity shown in Figure 8b, and to the formation of new chemical bonds responsible for absorption in the given wavelength range. Specifically, the absorption boundary shift towards the long-wave region is characteristic of an increase in the number of the conjugated double bonds (C–C=C–C=C–…) [57].

Since AFM data show no significant increase in surface roughness after plasma treatment, the increase in haze is more likely governed by chemical modification of the surface layers and the formation of bulk scattering centers, for example, due to structural inhomogeneity or micro-breakdowns. This interpretation is consistent with the PFM results, which show substantial reorganization of the ferroelectric domain structure during GDP poling. This may correlate with the PFM measurements, according to which poling using GDP increases the ferroelectric domain size from 86 nm to 552 nm.

## 4. Discussion

**Figure 10:**
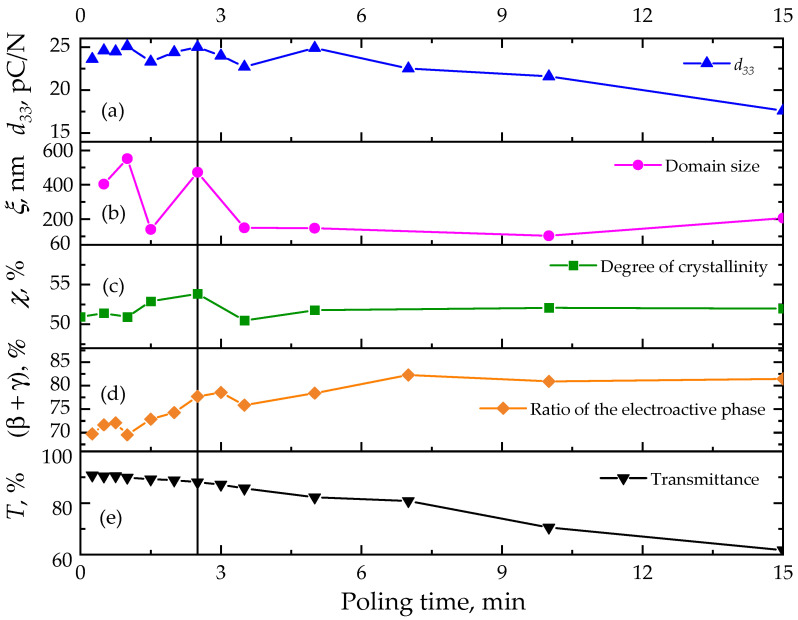
Dependencies of the piezoelectric coefficient (**a**), domain size (**b**), crystallinity degree (**c**), electroactive phase proportion (**d**), and light transmittance coefficient (**e**) on the poling time.

Figure 10 shows dependencies of the piezoelectric response *d*_33_, domain size *ξ*, crystallinity degree *χ_c_*, and electroactive phase fraction (*β* + *γ*) on poling time of the PVDF film samples in the glow-discharge plasma.

The piezoelectric coefficient *d*_33_ reaches high values within 1–5 min, exhibiting stepwise maxima in *d*_33_ observed at poling times of 1, 2.5, and 5 min. Beyond 5 min, the coefficient shows a nearly monotonic decrease.

In contrast, the degree of crystallinity varies weakly (≈52–56%). The proportion of the electroactive phase increases during the initial poling stage but approaches a quasi-plateau after approximately 3 min. This evolution in phase composition can be attributed to plasma-induced polymer recrystallization and the incorporation of amorphous phase chains into the ferroelectric domains formed under the applied electric field. This interpretation is supported by the pronounced evolution of the ferroelectric domain size: it increases from 86 nm to 552 nm within the first minute of poling, then abruptly decreases to 140 nm at 1.5 min, and reaches a second local maximum of 473 nm at 2.5 min. At longer processing times, the average domain size stabilizes in the range of approximately 100–210 nm.

The initial enhancement of *d*_33_ within the 1–5 min window is likely caused by the alignment of dipoles with the electric field and the consequent formation and growth of ferroelectric domains, accompanied by a slight rise in the electroactive phase content.

**Figure 11:**
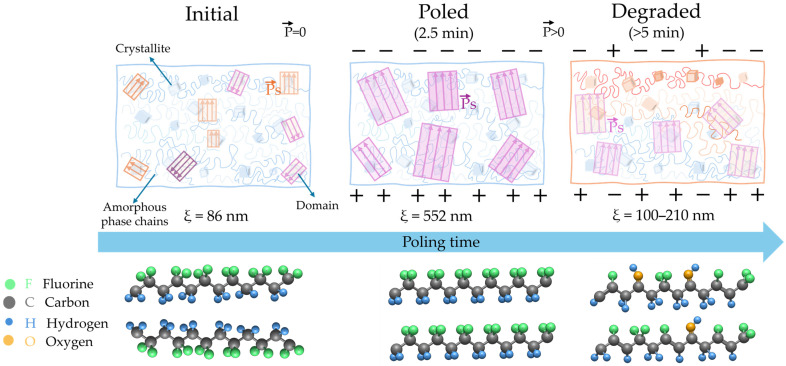
Schematic representation of the evolution of the ferroelectric domain structure, PVDF chain arrangement, and possible chemical modification during glow-discharge plasma poling.

The observed stepwise pattern may result from the non-uniform temporal progression of dipole orientation under the poling field [27]. In our opinion, the poling process proceeds in a non-linear manner because different mechanisms dominate at different stages of treatment. Within the initial period (0–1 min), the main contribution to the piezoelectric response arises from the orientation of dipoles of the electroactive phase and the growth of ferroelectric domains, whose size reaches 552 nm. This is followed by a rapid rearrangement of the phase structure of PVDF, during which the domain configuration changes and the average domain size decreases to 140 nm at 1.5 min. At 2.5 min, local maxima of the domain size (473 nm) and crystallinity degree (55.7%), together with an elevated electroactive phase content, are observed, accompanied by the next maximum of *d*_33_. Subsequently, the domain size decreases and approaches a plateau of ~100–210 nm, while *d*_33_ reaches another maximum at 5 min; at this stage, the dominant contribution is provided by the oriented dipoles of the electroactive phase, whose content approaches its plateau values (~0.78–0.82). Beyond 5 min, degradation of the near-surface layers prevails, resulting in a monotonic decrease in *d*_33_. A schematic representation of the evolution of the ferroelectric domain structure, PVDF chain arrangement, and possible chemical modification during glow-discharge plasma poling is presented in Figure 11.

It should be noted that the *d*_33_ values measured by the Berlincourt method reflect the macroscopic piezoelectric response, i.e., the integrated piezoelectric properties of the PVDF microstructure over a contact area of millimeter scale. Since this area exceeds the domain size by several orders of magnitude, the measured response is determined by the total poled volume rather than by the size of individual domains. It is therefore plausible that a larger number of small domains can generate a piezoelectric response comparable to that of a smaller number of large domains, provided that the total poled volume remains similar. This may explain why close *d*_33_ values (~25 pC/N) are observed for structurally different states of the film.

Further increasing the poling time beyond 5 min intensifies PVDF film degradation. This is evidenced by the decrease in *d*_33_, a significant deterioration in film transparency, and increased turbidity. These effects can be explained both by the reduction in domain size and, primarily, by chemical modification of the film’s surface layers, which likely involves the formation of conjugated double bonds.

According to the IR spectroscopy data, at a treatment time of 30 s, a peak characteristic of fluorinated ketones appears at 1720 cm^−1^ [58]. Apparently, this may be associated with the attack of ion radicals on the most vulnerable sites, namely head-to-head defects and –CH_2_–CH_2_– bonds. At the same time, after the initial appearance of the 1720 cm^−1^ band, its intensity practically does not increase at the early stages, which indicates the limited role of oxygen-containing radicals and suggests the participation of nitrogen radicals in hydrogen abstraction, especially since nitrogen is most likely unable to add to double bonds. At 90 s of treatment, a local peak at 1680 cm^−1^ is observed, presumably characteristic of terminal –CF_2_–COO– groups. During this time, complete scission of polymer chains at head-to-head defects with the formation of terminal carboxyl groups is likely to occur. Thus, short polymer chains combine into longer chains due to intermolecular dehydration. These processes likely affect the piezoelectric response of the films and contribute to the formation of maxima and minima in *d*_33_ (Figure 2).

At treatment times longer than 7 min, carboxyl groups are also associated with an increase in sample hydrophilicity (Figure 2), which is also consistent with the results reported in studies on plasma treatment of PVDF films [59,60]. Thus, it can be assumed that the decrease in contact angle at short treatment times occurs due to an increase in surface energy and the formation of polar groups during activation, whereas the further decrease is caused by the accumulation of carboxyl groups.

With an increase in treatment time to 10 and 15 min, a broad band at 1680 cm^−1^ appears in the IR spectra of the samples, presumably characteristic of terminal –CF_2_–COO– groups [61]. The increase in the number of terminal carboxyl groups may also be associated with a decrease in the molecular weight of the polymer, which is reflected in the increased brittleness of the samples after 15 min of treatment. It is also important to note that this trend is more pronounced in the ATR spectra, i.e., the near-surface layers are more strongly affected by changes in chemical composition.

Darkening of the samples at poling times longer than 5 min is probably associated with the formation of systems of conjugated double bonds, which have high extinction coefficients, up to 10^5^ dm^3^·mol^−1^·cm^−1^ [62], in the visible spectral range and exhibit a bathochromic shift in the absorption band with increasing conjugation length. Two mechanisms for the formation of such chromophores can be proposed. First, the formation of polyene sequences –(CH=CF)_n_– and –(CH=CH)_n_– as a result of plasma-induced dehydrofluorination of PVDF appears most likely, similar to processes observed during chemical dehydrofluorination by alkalis and amines [3,63]. Second, considering the participation of nitrogen radicals in the glow discharge in hydrogen abstraction, the formation of nitrogen-containing conjugated chromophores of the polymethine type cannot be excluded. In IR spectra, stretching vibrations of conjugated C=C bonds appear in the 1500–1600 cm^−1^ region with relatively low intensity and can be masked by strong absorption of carbonyl groups, whose band at 1680 cm^−1^ becomes noticeable only at long plasma treatment times.

This phenomenon warrants further investigation to develop strategies for preserving PVDF film transparency during plasma poling. Such strategies are particularly relevant for optical applications of PVDF films with ITO electrodes [64,65].

A comparative summary of GDP-based and conventional poling methods is presented in Table 4.

As seen, while the achievable piezoelectric response is comparable across different poling methods, GDP poling provides significant advantages in processing flexibility, vacuum compatibility, and the possibility of electrode-free operation on the film. In addition, this method offers improved process cleanliness and controllability.

However, in addition to requiring expensive and technically complex equipment compared with other poling methods, GDP poling has another limitation—the increased turbidity of PVDF films with prolonged plasma treatment, which is associated with surface modification processes. This drawback can be mitigated by optimizing the poling duration to balance piezoelectric and optical properties. Alternatively, pre-deposition of an electrode on the plasma-facing surface of the PVDF film can reduce surface degradation by limiting oxidation during poling.

From a technological perspective, the present results indicate that glow-discharge plasma poling offers several advantages beyond the activation of piezoelectricity itself. The electrode-free nature of the process eliminates the need for pre-deposited electrodes, while the vacuum environment allows subsequent electrode deposition without exposing the activated polymer surface to ambient atmosphere.

This feature may be particularly important for PVDF-based sensors and hydrophones, where the reliability of the polymer–electrode interface can strongly influence device performance. Fluoropolymers possess inherently low surface energy and often exhibit poor adhesion to metallic coatings. Plasma treatment is known to increase the surface energy of PVDF and improve the adhesion of subsequently deposited metal films. It was shown (Figure 2) that the water contact angle of the plasma-facing PVDF film surface decreases substantially already after 15 s of glow-discharge treatment, from ~85° to ~69°, and reaches ~40–45° after 3 min of treatment.

Consequently, GDP processing may simultaneously activate piezoelectricity, modify the polymer surface, and prepare it for subsequent vacuum metallization.

The ability to perform poling and electrode deposition within a single vacuum cycle may also reduce contamination of the activated surface by airborne particles, moisture, and organic contaminants. Such contamination may affect interfacial properties and the reproducibility of electrode formation, especially for thin metallic coatings.

These advantages may be particularly relevant for flexible ultrasonic sensors, hydrophones, and wearable piezoelectric devices operating under cyclic mechanical loading or elevated acoustic pressures, where electrode reliability represents a critical factor determining long-term device performance. In hydrophone applications operating under high acoustic pressures, the integrity of the electrode layer may become a limiting factor affecting measurement stability and service life. In some measurement and calibration procedures, conductive coatings may require periodic renewal, which complicates operation. Therefore, vacuum-compatible processing routes combining poling and electrode deposition may provide additional advantages for such applications.

Although the present work focuses on the fundamental relationship between plasma treatment, structural evolution, and piezoelectric response, future studies should address the stability of plasma-induced surface modification and the long-term durability of electrodes deposited after GDP treatment. Such studies may further establish GDP poling as an integrated technological step combining poling and electrode formation.

## 5. Conclusions

The results of PVDF film poling experiments using glow-discharge plasma demonstrate the high efficiency of this method. GDP poling enables a 25 μm thick PolyK PVDF film, without pre-deposited electrodes, to achieve a side-averaged piezoelectric response of up to 25 pC/N, with a narrow confidence interval of approximately ±0.7 pC/N, within 1–5 min of poling and without requiring additional heating. In addition to the enhancement of the piezoelectric response, plasma treatment substantially increases the wettability of the plasma-facing PVDF film surface, reducing the water contact angle from approximately 85° to 42° within 3 min of treatment. When plasma poling is integrated with subsequent vacuum magnetron deposition of thin-film electrodes, this plasma-induced surface activation may become an additional technological advantage of the process by promoting improved adhesion of the deposited metal layer. Such integration may simplify the fabrication workflow, reduce sample handling, and improve the cleanliness and overall efficiency of the manufacturing process.

The results suggest that the initial increase in piezoelectric activity within 1–5 min is not mainly caused by changes in crystallinity, but rather by reorganization of the domain structure. This is accompanied by an increase in the electroactive phase fraction and, more importantly, by a pronounced non-monotonic evolution of the ferroelectric domain size (from 86 nm up to 552 nm within the first minute of poling) as dipoles align with the external field. 

The stepwise change in the piezoelectric response *d*_33_, with three distinct maxima over time, suggests that dipole rotation during GDP poling does not follow a simple linear pathway. Instead, the data point to a staged reorientation of polymer chains and domain structure. It is also important that similar *d*_33_ values are obtained for samples with different structural states, indicating that more than one structural configuration can produce a comparable macroscopic electromechanical response.

However, extending the treatment beyond 5 min leads to degradation of both the bulk PVDF structure and the surface layer, as indicated by a monotonic decrease in *d*_33_, and a deterioration of optical properties (increased haze and turbidity). The primary cause of this degradation is likely chemical modification and breakdown of the polymer surface layers, accompanied by the formation of conjugated double bonds.

Therefore, this study demonstrates that near-maximum piezoelectric activity can be achieved within an optimal poling-time window of 1–5 min. Samples poled for different durations exhibit comparable *d*_33_ values (~25 pC/N), while differing in phase composition, domain structure, and optical properties. Prolonged treatment beyond 5 min initiates competing degradation processes that negatively affect the PVDF film properties. When optical clarity is critical, the poling time should be optimized to balance piezoelectric response and haze.

These findings highlight the potential of glow-discharge plasma poling as a technologically relevant approach for scalable fabrication of PVDF-based flexible piezoelectric sensors and integrated polymer electronic devices.

Future work should focus on device-level validation of the proposed approach, including electrode deposition after GDP poling, cyclic mechanical loading, bending stability, long-term retention of piezoelectric response, and evaluation of the output signal in flexible piezoelectric sensor configurations.

## Figures and Tables

**Table 1:** Phase content calculations based on FTIR data (transmission mode).

t, min	Initial	0.5	0.75	1	1.5	2	2.5	3	3.5	5	7	10	15
(*β* + *γ*) content	0.77	0.69	0.72	0.69	0.73	0.74	0.78	0.79	0.75	0.78	0.82	0.80	0.81
I*_α_*/I_am_ peak ratio	1.07	1.21	0.99	1.17	0.89	0.84	0.71	0.67	0.81	0.68	0.54	0.69	0.51

**Table 2:** DSC data.

t, min	Initial	0.5	1	1.5	2.5	3.5	5	10	15
Melting enthalpy ΔHm, J/g	51.8	53.7	53.2	55.3	56.3	52.3	54.1	54.4	54.3
Electroactive phase content (FTIR)	0.77	0.69	0.69	0.73	0.78	0.75	0.78	0.8	0.81
Crystallinity degree *χ_c_*, %	52.7	53.6	53.1	54.9	55.7	52.3	53.5	53.7	53.5

**Table 3:** Piezoresponse force microscopy data.

t, min	0	0.5	1	1.5	2.5	3.5	5	10	15
RMS, nm	27	36	33	27	32	35	24	26	29
ξ, nm	86	404	552	140	473	150	147	103	206

**Table 4:** Comparison of PVDF film poling methods.

Parameters	Glow-Discharge Plasma	Corona Discharge	Contact
Achievable *d*_33_ for pure PVDF film, pC/N	Comparable (up to ~25 pC/N)	Comparable (up to ~25 pC/N)	Comparable (up to ~25 pC/N)
Processing time, min	1–5	1–30 [66]	1–60 [67]
Homogeneity of poling field	High, owing to flat electrodes and vacuum environment	Low, owing to highly localized electric field near point/edge electrodes [45]	High, but limited by electrode–film interface quality [68]
Effect of dielectric breakdown	Local surface damage in breakdown regions	Structural damage and reduced piezoelectric response in affected regions [29]	Structural damage and possible short-circuiting in breakdown regions [69]
Flexibility of electrode configuration on film	Supports electrode-free, single-sided, and double-sided configurations [43]	Limited: single-sided configuration with a counter electrode [45]	Limited: electrodes on both sides are required (sandwich configuration) [69]
Process cleanliness	Clean, vacuum-based; no liquid dielectric media required	Clean (no direct contact), but exposure to ambient species may affect activated surfaces	Requires liquid dielectric media (e.g., oil) [67], risk of contamination
Compatibility with vacuum deposition of electrodes in one cycle	Potentially compatible with in situ vacuum deposition	Not compatible	Not compatible
Availability of roll-to-roll film poling	Potentially available	Available	Not available
Effect on optical properties	Increased turbidity after prolonged plasma treatment occurs if transparent electrode on plasma-facing surface is not pre-applied	No significant changes reported or possible surface activation effects	No changes
Thermal film deformation	May occur after prolonged plasma treatment	May occur during prolonged heating	May occur during prolonged heating
Processing equipment costs	Requires a vacuum chamber and pumping system; relatively high equipment cost	Requires a corona discharge unit; moderate cost	Relatively simple and low-cost setup; high-voltage power supply required

## Data Availability

The original contributions presented in this study are included in the article. Further inquiries can be directed to the corresponding author.
